# Diabetic Retinopathy in Type 2 Diabetes Mellitus Patients Attending the Diabetic Clinic of the University of Gondar Hospital, Northwest Ethiopia

**DOI:** 10.1155/2021/6696548

**Published:** 2021-03-31

**Authors:** Asamere Tsegaw, Shitaye Alemu, Abere Dessie, Christopher C. Patterson, Eldryd H. O. Parry, David I. W. Phillips, Elisabeth R. Trimble

**Affiliations:** ^1^Department of Ophthalmology, College of Medicine and Health Sciences, University of Gondar, Gondar, Ethiopia; ^2^Department of Internal Medicine, College of Medicine and Health Sciences, University of Gondar, Gondar, Ethiopia; ^3^Centre for Public Health, Queen's University Belfast, Belfast, UK; ^4^London School of Hygiene and Tropical Medicine and Tropical Health Education Trust, London, UK; ^5^MRC Life Course Epidemiology Unit, University of Southampton, Southampton, UK

## Abstract

**Objective:**

To investigate the level of diabetic retinopathy in type 2 diabetes (T2DM) patients attending the University of Gondar Hospital (UGH) Diabetic Clinic, Northwest Ethiopia.

**Methods:**

An audit was carried out involving a total of 739 T2DM patients attending at the diabetic clinic of UGH. They represented approximately 90% and 50% of all T2DM patients under regular review at the urban and rural diabetic clinics of UGH, respectively. All were supervised by the same clinical team for a long period. Eye examinations were performed for visual acuity, cataract, and retinal changes (retinal photography and slit-lamp biomicroscopy). Body mass index (BMI) and HbA1c levels were measured. The presence or absence of hypertension was recorded.

**Results:**

Men constituted 41.5% of the group, the mean age at diagnosis of T2DM was 50.4 years, and 50.2% were hypertensive. The BMI was 25.0 ± 4.1 kg/m^2^, and HbA1c was 7.75 ± 1.63% (61.2 ± 17.8 mmol/mol) (mean ± SD, for BMI and HbA1c)). Severe visual impairment/blindness was reported in 10.6%, 15.2% had cataract, 16.0% had retinopathy, and 11.1% had maculopathy. The prevalence of retinopathy increased with time from diagnosis of T2DM (chi-square for trend, *p* < 0.001) and with increasing HbA1c level (chi-square for trend, *p*=0.03).

**Conclusion:**

These results compare well with the most recent results in well-equipped, wealthier regions of the world and show the importance of stable healthcare infrastructure for chronic-disease management.

## 1. Introduction

Sub-Saharan Africa has been facing an epidemic of T2DM driven by urbanization, lifestyle changes, poor diet, smoking, other environmental factors, and ageing. The International Diabetes Federation has estimated that the number of adults with diabetes in Africa will increase from 19.4 million in 2019 to 47.1 million in 2045 [1].

Diabetes causes significant morbidity, disability, and early mortality. The diabetes epidemic, therefore, poses significant health and socioeconomic challenges for a continent simultaneously facing other health challenges including infectious diseases.

Diabetes causes visual impairment through early-onset cataract and diabetic retinopathy, a progressive disease of the retinal microvasculature. Out of the 39 million people with blindness from various eye diseases globally, in 1.8 million (4.8%), it was a result of diabetic retinopathy [2].

The epidemiology of diabetic retinopathy in Africa has been systematically reviewed [3]. In population-based studies, the reported prevalence range in patients with diabetes for diabetic retinopathy was 30.2 to 31.6%, proliferative diabetic retinopathy 0.9 to 1.3%, and any maculopathy 1.2 to 4.5%. In diabetes clinic-based surveys, the reported prevalence range for diabetic retinopathy was 7.0 to 62.4%, proliferative diabetic retinopathy 0 to 6.9%, and any maculopathy 1.2 to 31.1% [3].

There are few reports on the prevalence of diabetic retinopathy among Ethiopian diabetic patients; most of these reports were published several decades ago, and therefore, there is a need for more recent information. The previous studies depended on direct ophthalmoscopic examination, with its inherent weaknesses, to diagnose diabetic retinopathy. Second, there is now a proven link between glycemic control and microvascular disease of the retina [4, 5]; it is time, therefore, to evaluate the effect of glycemic control on diabetic retinopathy in Ethiopia.

In this audit report, we used retinal photography and stereoscopic examination with slit-lamp microscopy to assess the visual problems associated with diabetes, such as cataract and retinopathy, in a group of patients with T2DM attending diabetic clinics in Northwest Ethiopia.

## 2. Patients and Methods

### 2.1. Study Design and Period

The audit was conducted from March 2017 to February 2018 at the University of Gondar comprehensive specialized hospital involving diabetic patients on follow-up for routine diabetic care at diabetic clinics associated with the university hospital.

### 2.2. Study Area

The University of Gondar is one of Ethiopia's main medical universities, and its comprehensive specialized hospital is one of the major teaching hospitals in the country. It is a referral centre for specialized medical care for the entire region of Northwest Ethiopia. Gondar is one of the centres where there has been a sustained development of infrastructure to manage chronic diseases (diabetes, hypertension, cardiac diseases, etc) in a way that effectively includes both urban and rural dwellers, through a network of urban and rural clinics centred on Gondar University Hospital. The diabetes care service is part of the chronic disease management program; routine diabetes care has been supervised by the same care team at both the central and rural clinics for more than 20 years.

### 2.3. Study Population

All patients with T2DM attending the Gondar University Hospital diabetic clinic and the associated rural clinics were invited to attend the central clinic for assessment of their sight, including retinal photography with a static camera and slit-lamp microscopy. This report is on 739 patients who represent approximately 90% of urban and 50% of rural patients who attend the diabetes clinics regularly.

### 2.4. Data Collection Procedure

#### 2.4.1. Visual Acuity

Tumbling-E Snellen's charts were used to assess visual acuity, starting the examination with the patient at 6 m distance from the chart in a well-illuminated room. Best-corrected visual acuity was measured for both eyes, and grading of visual impairment and blindness was performed according to the WHO classification system [6] as follows: visual acuity better or equal to 6/18- normal; visual acuity less than or equal to 6/24 and better than 6/60- moderate visual impairment; visual acuity less than or equal to 6/60 and better than counting fingers at 3 m- severe visual impairment; and visual acuity less than or equal to counting fingers at 3 m- blindness; the results for the eye with the best-corrected visual acuity were recorded.

#### 2.4.2. Cataract

Cataract was diagnosed by examination with a slit-lamp microscope by a senior ophthalmologist with subspecialty training in vitreoretinal conditions.

#### 2.4.3. Retinopathy and Maculopathy

A Top Con TRC-NW65 Non-Mydriatic Retinal Camera (Topcon Medical Systems Inc, Oakland, New Jersey) was used for retinal photography. This was carried out under standardized conditions in a darkened room after the eyes were treated with 1% tropicamide (although this camera can operate without mydriasis); two photographs were taken for each eye, one centred on the optic disc and one centred on the macula. Grading of the retinal changes was made using the Diabetic Retinopathy Study guidelines and recorded in six categories: mild, moderate, and severe nonproliferative retinopathy and early, high risk, and advanced proliferative retinopathy [7]. Maculopathy was diagnosed when there were hard exudates on the macula and/or thickening obvious on slit-lamp examination. Slit-lamp examination was carried out only if there was evidence of retinopathy or suspicion of cataract not visible to the naked eye. All retinal photography was carried out by a single, trained senior nurse. All retinal photographs with signs of retinopathy were cross checked and graded by a senior ophthalmologist with subspecialty training in vitreoretinal conditions.

### 2.5. Hemoglobin A1c (HbA1c)

HbA1c measurements were made on venous blood, using cartridges containing boronate affinity columns from Bio-Rad, in a Bio-Rad in2it A1c analyser for point-of-care testing, according to the manufacturer's instructions (Bio-Rad Laboratories Inc, Hercules, California). Quality control was carried out at least once per day and/or after the analyzer was moved.

### 2.6. Patient Management

Management of all patients is affected by the limited laboratory resources available to monitor progress; thus, HbA1c, microalbuminuria, and lipids are not measured as a routine. Alterations in disease management are based on the recent clinical history, on fasting blood glucose levels measured at the clinic visit, and the presence or absence of hypertension (≥140/90 mm Hg).

Most were treated only with oral hypoglycemic agents; however, 178 were subsequently changed to insulin and 3 had a combination of insulin and oral hypoglycemic agents at the time of this cross-sectional study. The initial choice of the oral hypoglycemic agent was determined by BMI at presentation: glibenclamide for those with BMI <27 kg/m^2^ and metformin if BMI >27 kg/m^2^. Glibenclamide was increased in increments of 5 mg/day to a maximum of 20 mg/day, and metformin was added if indicated; metformin was increased to a maximum of 2 g/day after which glibenclamide was added; the next addition was basal insulin if required. However, when poor glycemic control persisted, oral hypoglycemic agents were removed and treatment was changed to insulin alone.

### 2.7. Blood Pressure Control

The aim was to maintain a blood pressure <140/90 mm Hg. The choice of antihypertensive drugs was limited to hydrochlorothiazide, enalapril, and nifedipine, alone or in combination. Most patients were controlled with a combination of two drugs.

### 2.8. Statistical Analyses

Statistical analyses were performed using SPSS version 21 (IBM Corp, Armonk, New York). Baseline results are presented as mean ± SD or percentages, and comparisons between groups were made using the *t*-test, chi-square test/Fisher's exact probability test, or the chi-square test for trend.

## 3. Results

### 3.1. Demography

A total of 739 T2DM patients were entered into the audit, of these 41.5% were men. The average age at the time of diagnosis was 50.4 years, the average time elapsed since diagnosis was 5.9 years, and the mean age at screening was 56.3 years (Table 1).

Most patients fell into 4 occupational groups: housewives (*n* = 276), government employees (*n* = 235), merchants (*n* = 92), and farmers (*n* = 15). The demographics of government employees were similar in the urban and rural areas with respect to most measured parameters; however, the age at diagnosis (urban 46.0 ± 8.4 yr and rural 47.8 ± 6.2 yr) tended to be lower than for the T2DM group as a whole, 50.4 ± 10.7 years (mean ± SD). By contrast, the small group of farmers (*n* = 15) had the oldest age at diagnosis (55.6 ± 10.3 years).

### 3.2. Body Mass Index (BMI)

The mean BMI of the total group was 25.0 kg/m^2^ (Table 1); of these, a total of 54.2% were less than 25 kg/m^2^, and 4.8% were less than 18.5 kg/m^2^. The BMI of male patients was lower than female patients (24.4 ± 3.3 versus 25.5 ± 4.6 kg/m^2^, *p* < 0.001). Although the farmers (*n* = 15) had the lowest BMI (21.1 ± 2.6 kg/m^2^) (mean ± SD), there was no significant difference between the urban and rural groups as a whole (not shown).

### 3.3. HbA1c

The average HbA1c level was 7.75% (61.2 mmol/mol) (Table 1).

In the 369 (50%) patients with data available, there were no significant differences in the average HbA1c levels between urban and rural groups, nor between male and female patients (not shown). Mean HbA1c levels in 36.0 percent patients were ≤7% (≤53 mmol/mol); 45.0 percent were 7.1–9.0% (54–75 mmol/mol); and 19.0 percent were ≥9.1% (≥76 mmol/mol).

### 3.4. Treatment Groups: Oral Agents and Insulin Treatment

Although all patients commenced treatment with oral hypoglycemic agents, 178 were later moved to treatment with insulin alone. The age of onset of the insulin-treated group was lower (48.3 ± 10.7 vs. 51.0 ± 10.7 years, *p* < 0.003) and BMI was also lower (24.4 ± 4.0 vs 25.2 ± 4.2 kg/m^2^, *p* < 0.03) than the group that remained on oral hypoglycemic agents.

### 3.5. Eye Examination

#### 3.5.1. Visual Acuity

Visual acuity was assessed in 719 (97.3%) of the 739 patients. In those who were tested, 73.6% had normal visual acuity, 15.9% had moderate visual impairment, and a total of 10.6% had either severe visual impairment or were blind (Table 2).

#### 3.5.2. Cataract, Retinopathy, and Maculopathy

(1) *Cataract*. Cataract at various levels of development was present in 112 (15.2%) cases. Prevalence of cataract increased with the time from diagnosis (Table 3(a)) and with the age of the patient at screening (Table 3(b)).

(2) *Retinopathy*. Interpretable (gradable) retinal photographs were obtained in 673 (91.1%) of the 739 patients, which included several patients with some degree of cataract development. Retinopathy was present in 108 cases (16.0%). Retinopathy was nonproliferative in 103 (15.3%) cases and proliferative in 5 (0.7%) cases (Table 4; see footnote for details). As expected, the prevalence of retinopathy increased with the time from diagnosis, increasing from 9.3% when the duration was 5 years or less, to 16.4% for those 5–10 years from diagnosis and 41.6% when the time from diagnosis was 11 or more years (chi-square test for trend, *p* < 0.001).

In contrast to cataract, retinopathy did not increase with the age of the patient (chi-square test for trend, *p*=0.24). HbA1c was measured in 335 (45.3%) patients who had interpretable retinal photographs (total, *n* = 673). Retinopathy was present in 10.1 per cent patients with HbA1c ≤ 7% (≤53 mmol/mol), in 16.6 per cent patients with HbA1c 7.1–9.0% (54–75 mmol/mol), and in 21.5 per cent patients with HbA1c ≥ 9.1% (≥76 mmol/mol) (chi-square test for trend, *p*=0.03).

Maculopathy was recorded in 75 (11.1%) of the 673 cases with interpretable retinal photographs. The prevalence of maculopathy increased with time from diagnosis of diabetes (chi-square test for trend, *p* < 0.001), being 6.9% at 5 years or less,12.0% after 6–10 years, and 25.7% at 11 or more years from diagnosis (Table 4). Maculopathy was present in 33.3%, 79.2%, and 94.1% of patients with mild, moderate, and severe nonproliferative retinopathy and in all cases with proliferative retinopathy (not shown). No case of isolated maculopathy was recorded.

### 3.6. Comorbidities

#### 3.6.1. BMI and Hypertension

There was a positive association between BMI in the categories <18.5 kg/m^2^, 18.5–24.9 kg/m^2^, 25.0–29.9 kg/m^2^, and 30+ kg/m^2^ and the diagnosis of hypertension, which rose from 37.1% to 46.7%, 55.9%, and 54.5% in these BMI categories, respectively. The overall percent with hypertension in this group of 726 patients was 50.3% (Table 5(a)).

#### 3.6.2. BMI and Retinopathy

There was an inverse association between BMI and the presence of retinopathy (Table 5(b)).

#### 3.6.3. Hypertension and Retinopathy

In the 673 patients who had both their blood pressure measured and whose retinal photographs were gradable, there was no association between retinopathy and the presence of hypertension (Table 6). Of the 108 patients with retinopathy, 45(41.7%) had hypertension compared with 283(50.0%) of the 565 patients without retinopathy (*χ*^2^ = 2.6, *p*=0.11).

## 4. Discussion

This report is part of an audit undertaken to assess the visual problems and related disabilities in patients suffering from diabetes who live in Gondar and the surrounding region of Northwest Ethiopia and refers only to those patients whose initial treatment did not include insulin, type 2 diabetes. In this region of Northwest Ethiopia, the overall direction, ethos, goals, and management of both the urban and rural clinical diabetes services, including patient education, have been overseen by a stable, experienced clinical team lead by the same consultant physician for more than 20 years.

In this study, we investigated the occurrence of diabetic retinopathy and its determinants among subjects with T2DM from mixed rural and urban areas attending the diabetic clinic for routine diabetes care. We found the prevalence of diabetic retinopathy, diabetic maculopathy, and proliferative diabetic retinopathy among them to be 16.0%, 11.1%, and 0.7%, respectively.

In a previous publication from this region on type 1 diabetes (T1DM), we compared urban and rural groups and found urban dwellers had a significantly higher prevalence of retinopathy compared to rural patients, 16.1% versus 5.0%, and the overall prevalence of diabetic retinopathy in T1DM patients was 8.6%. [8]. The prevalence of diabetic retinopathy in this report among T2DM patients, 16%, is higher than the prevalence of 8.6% we reported in T1DM patients [8] but is much lower than that in previous similar diabetic clinic-based reports from Ethiopia, 37.8% [9], 41.4% [10], and 41.1% [11]. It is also lower than that of diabetic clinic-based studies from other African countries, which range from 26.5% to 62.4% [12–17]. Population-based studies of diabetic retinopathy in Africa that used retinal photography as a means of diagnosis report prevalence of 30.2% in Mauritius [18] and 15.7%, 31.6%, and 41.5% in Egypt [19–21]. The result in our study, 16.0%, is lower than results in most of these reports.

However, the rate of maculopathy in our study, 11.1%, is higher than the 6% reported from Jimma in Ethiopia [10] and 4.5% for a population-based study in Egypt [19]. The rate of maculopathy in this study is comparable with diabetic clinic-based African reports of 10% and 15.1% in South Africa [13], 15% in Malawi [17], and 11.5% in Egypt [20]. The fact that the two-dimensional retinal photograph was supplemented with stereoscopic examination of the retina with slit-lamp microscopy to diagnose diabetic macular edema may have increased the sensitivity of diagnosing maculopathy in our study as all patients with retinopathy on retinal photography were re-examined with slit-lamp microscopy.

The prevalence of proliferative diabetic retinopathy in the previous Ethiopian reports ranged from 1.7% to as high as 9.9% [9–11, 22, 23], which were higher than the estimate of 0.7% in our patients. Our estimate is lower than in reports in population-based studies from Africa, 1.3% in Mauritius [18] and 0.9% in Egypt [19], and much lower than in other diabetic clinic-based African studies, 6.1% and 5.6% in South Africa [13, 24], 5.7% and 7.3% in Malawi [16, 17], and 2.3% in Egypt [21].

The association between the duration of diabetes and the prevalence of retinopathy is very well known [9, 20, 21, 25] as also found in this study. The rate of retinopathy also increased with increasing level of HbA1c. In the Korea National Health and Nutrition Examination Survey, the prevalence was (approximately) 10% at less than 3 years, 12% at 3–10 years, and 31% at greater than 10 years [26]. In rural Korea, the incidence was 20.2% at 1–5 years, 23.2% at 5–10 years, and 52.3% at greater than 10 years [27]. In Spain, the prevalence of retinopathy after 9 years in a cohort of T2DM patients was 28% [28]. The results from the present study in Northwest Ethiopia, therefore, compare favourably with results from these studies which were undertaken in wealthier countries.

The mean HbA1c level in our study was 7.75%, and this is much lower than in previous Ethiopian reports, 10.4% [11], 11.3% [23] and studies reporting 11.3%, and 9.2% in South Africa [13, 29]. The prevalence of retinopathy increased, as expected, with increasing levels of HbA1c, in agreement with many similar studies [17, 21, 25, 29, 30]. In recent years, clinics have set goals for a much tighter control of blood glucose levels following the publication of results from the UK Prospective Diabetes Study [4, 5] in T2DM. Almost certainly, tighter glycemic control coupled with a stable network of urban and rural clinics has resulted in the reduced levels of diabetic retinopathy in the present report.

The negative association of BMI with retinopathy in the present group of patients was counterintuitive because of the often-reported, positive relationship between BMI and insulin resistance and BMI and hypertension [31, 32]. However, it should be pointed out that, in two other published studies, a negative relationship between BMI and the development of retinopathy in T2DM has been recorded [26, 33] while a more recent meta-analysis has failed to demonstrate an association [34]. Most studies have come from well-nourished, urbanized populations; there have been very few studies in poor rural areas as in Gondar where BMI levels are low [8]. It was noted that those with the lowest BMI were among those that eventually required insulin treatment.

It is well known that there is a high prevalence of hypertension in diabetes (20–60%) [35–37], and hypertension is associated with the development of diabetic retinopathy in patients with T2DM [4]; although half our case series had hypertension, no statistically significant association between retinopathy and hypertension was shown in the present study (Table 6). Again, there is a paucity of data on determinants of retinopathy (microvascular disease) among diabetics in resource-poor countries and, in particular, little available data in populations such as in Northwest Ethiopia where the prevalence of atherosclerotic (macrovascular) disease is low; we do not have a complete explanation for this difference. It may be due to different underlying relationships between retinopathy and blood pressure in this population or as a result of the high degree of blood pressure control in the majority of patients. At any one time, 75% patients treated for hypertension had satisfactory blood pressure levels (<140/90 mmHg). Others have shown that adequate control of blood pressure can reduce the effect of hypertension on the development of retinopathy [5].

Visual acuity was severely affected in a sizeable proportion of cases with 76 (10.6%) study patients being either severely visually impaired or blind; cataract was diagnosed in 15.2% of patients. Reports of visual impairment and blindness from previous similar studies range from 1.4% [17] to 31% [3]. In countries such as Ethiopia, blindness is a factor with associations to poverty; in those cases with cataract and no retinopathy, cataract removal is now an accessible procedure with life-changing outcomes.

## 5. Conclusions

This audit has shown that the prevalence of diabetic retinopathy at the University of Gondar hospital diabetic clinic is lower than in previous reports from Ethiopia and other similar studies from African healthcare facilities and compares well with reports of the most recent studies from countries with wealthier economies [38–40]. This shows the importance of a stable healthcare infrastructure for chronic-disease management in the healthcare setting of a developing country.

## Figures and Tables

**Table 1:** Demographic and clinical characteristics of T2DM patients.

Total number	739
Male gender, *n* (%)	307 (41.5)
Age at diagnosis, years^*∗*^	50.4 ± 10.7 (range 26.0 – 85.0)
Time since diagnosis, years^*∗*^	5.9 ± 5.1 (range 0.1 – 30.0)
Age at screening, years^*∗*^	56.3 ± 10.7
BMI, kg/m^2^ (*n*)^*∗*^	25.0 ± 4.1(726)
HbA1c^*∗*^ (%) (*n*) (mmol/mol)	7.75 ± 1.63 (369) [61.2 ± 17.8]
Hypertensive *n* (%)	371 (50.2)

^*∗*^Mean ± SD.

**Table 2:** Visual acuity level of T2DM patients.

Total tested, *n*^a^	719
6/6 – 6/18 (normal), *n* (%)	529 (73.6)
6/24 – 6/60 (MVI)^b^, *n* (%)	114 (15.9)
≤6/60 – 3/60 (SVI)^c^, *n* (%)	32 (4.5)
≤3/60 (blind), *n* (%)	44 (6.1)

^a^Visual acuity results were not available for 20 patients. ^b^MVI, moderate visual impairment. ^c^SVI, severe visual impairment.

**Table 3:** Prevalence of cataract in T2DM patients.

(a) Time since diagnosis (yr)	≤5	6 – 10	11+	Total
Number of patients	413	207	119	739
Prevalence of cataract, *n* (%)	43 (10.4)	40 (19.3)	29 (24.4)	112 (15.2)
(b) Age at screening (yr)	18–40	41–60	61+	Total
Number of patients	49	464	226	739
Prevalence of cataract, *n* (%)	2 (4.1)	44 (9.5)	66 (29.2)	112 (15.2)

Time since diagnosis vs. cataract, *p* < 0.001 (chi-square test for trend). Age at screening vs. cataract, *p* < 0.001 (chi-square test for trend).

**Table 4:** Prevalence of retinopathy and maculopathy with time since diagnosis among T2DM patients.

	Time since diagnosis (years)			
	≤5	6 – 10	11+	Total
Number of patients^a^	389	183	101	673
Retinopathy, *n* (%)^b^	36 (9.3)	30 (16.4)	42 (41.6)	108 (16.0)
Maculopathy, *n* (%)	27 (6.9)	22 (12.0)	26 (25.7)	75 (11.1)

^a^Number of patients with interpretable retinal photographs. ^b^Severity of retinopathy—nonproliferative (*n*): 33 mild, 53 moderate, 17 severe; proliferative (*n*): 1 early, 4 advanced. Time since diagnosis vs. retinopathy, *p* < 0.001 (chi-square test for trend). Time since diagnosis vs. maculopathy, *p* < 0.001 (chi-square test for trend).

**Table 5:** Association of BMI with (a) hypertension and (b) retinopathy among T2DM patients.

	BMI (kg/m^2^)				
	<18.5	18.5 – 24.9	25.0 – 29.9	30+	Total
(a) Hypertension					
Number of patients^a^	35	360	254	77	726
Hypertension, *n* (%)^b^	13 (37.1)	168 (46.7)	142 (55.9)	42 (54.5)	365 (50.3)
(b) Retinopathy					
Number of patients^c^	30	320	237	73	660
Retinopathy, *n* (%)^d^	12 (40.0)	51 (15.9)	35 (14.8)	8 (11.0)	106 (16.1)

^a^Patients in whom BMI and blood pressure were measured. ^b^BMI vs. hypertension, *p*=0.03 (chi-square test for trend). ^c^Patients with retinal photographs and measured BMI. ^d^BMI vs. retinopathy, *p*=0.01 (chi-square test for trend).

**Table 6:** Association between the presence of hypertension and retinopathy in the 673 patients who had their blood pressure measured and gradable retinal photographs.

Diabetic retinopathy	Blood pressure status		Total
	Normotensive	Hypertensive	
Absent, *n* (%)	282	283 (50.1)	565
Present, *n* (%)	63	45 (41.6)	108
Total	345	328	673

## Data Availability

All data are fully available without restriction on request.
